# Response to “Adjuvant vitamin C in cardiac arrest patients undergoing renal replacement therapy: an appeal for a higher high-dose”

**DOI:** 10.1186/s13054-018-2200-0

**Published:** 2018-12-19

**Authors:** Angelique M. E. Spoelstra – de Man, Harm-Jan de Grooth, Paul W. G. Elbers, Heleen M. Oudemans – van Straaten

**Affiliations:** Department of Intensive Care Medicine, Amsterdam UMC, Vrije Universiteit Amsterdam, De Boelelaan 1117, 1081 HV Amsterdam, The Netherlands

**Keywords:** Vitamin C, Cardiac arrest, Pharmacokinetics, Continuous renal replacement therapy

We thank Honore et al. [1] for raising the issue of the optimal dose of vitamin C in cardiac arrest patients, especially those requiring continuous renal replacement therapy (CRRT). They propose to treat patients post-cardiac arrest with 6 g daily. However, the efficacy of vitamin C after cardiac arrest has not been settled yet, let alone the optimal dose. Up to now, only one small trial in septic patients compared two different doses: 200 mg/kg/day (~ 16 g/day) seemed superior to 50 mg/kg/day (~ 4 g/day) [2]. All other studies in varying populations (but not after cardiac arrest) investigated a single dose (3 g up to 125 g/day). So, clinical studies on efficacy and dose in the cardiac arrest population are crucial before recommending an optimal dose. We are starting such a study (NCT03509662).

Honore et al. propose to double this dose to 12 g during CRRT based on three studies. Two of these studies included patients on intermittent chronic hemodialysis/diafiltration. One found a mean loss of 66 mg vitamin C per day (200 mg/week) [3], the other did not report total loss. The only study in patients on CRRT (continuous venovenous hemofiltration (CVVH)) reported a median loss of 93 (0–372) mg vitamin C per day. Furthermore, the mean plasma concentration in the CRRT patients was not lower than in the contemporary ICU population (43 (23–57) μmol/L vs 37 (28–108) μmol/L) [4]. This suggests that not the CRRT but the critical illness is the cause of the low vitamin C concentrations.

We calculated vitamin C loss by CVVH in a patient from our pharmacokinetic study treated with 1 g intravenous vitamin C twice daily [5]. He had an average vitamin C plasma concentration of 17.3 mg/L (98.6 μmol/L) during the 48-h treatment period (AUC 832 mg/L∙48 h). Simultaneous plasma and post-filter measurements confirmed a sieving coefficient of about 1. At an effluent CVVH flow of 2 L/h, this amounts to a total loss by CVVH of 830 mg per day (17.3 mg/L∙2 L/h∙24 h), 41% of the administered dose. Removal by CRRT is therefore lower than removal by the native kidney (1476 mg/day; 74% of administered dose) in the four other patients not on CVVH treated with 2 g/day bolus infusions. The reason is that a CVVH dose of 2 L/h corresponds to a clearance of 33 ml/min, much lower than a native kidney clearance. The plasma vitamin C concentrations of this patient (patient 5; Fig. 1) were all within the normal range, suggesting that a dose of 1 g vitamin C twice a day may be sufficient to maintain normal plasma concentrations during CVVH.

**Fig. 1:**
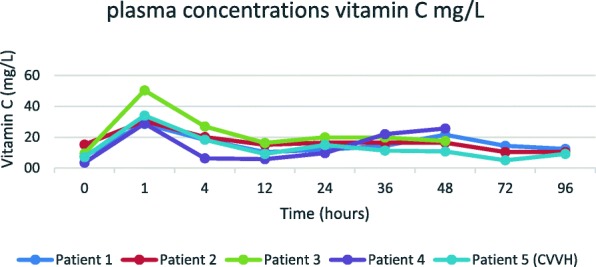
Plasma concentrations of vitamin C in five patients treated with 1 g intravenous vitamin C twice daily. Patient 5 was treated with CVVH

In conclusion, vitamin C loss by CVVH does not seem to be higher than loss by the native kidney. So based on the scarce available data, it is not necessary to increase the vitamin C dose during CVVH above 2 g/day when normal plasma concentrations are targeted. Whether higher concentrations are beneficial needs to be shown. In our RCT in cardiac arrest patients more pharmacokinetic data will be collected.

## Abbreviations

CRRT: Continuous renal replacement therapy
CVVH: Continuous venovenous hemofiltration
